# Supplementary material for: Constructing the Sulfur-Doped CdO@In2O3 Nanofibers Ternary Heterojunction for Efficient Photocatalytic Hydrogen Production
Source: Nanomaterials (Basel). 2023 Jan 18;13(3):401. doi: 10.3390/nano13030401 (PMC9920990; doi:10.3390/nano13030401)
Supplement: Supplementary file 1 [file nanomaterials-13-00401-s001.zip › nanomaterials-2136201-supplementary.pdf]

nanomaterials

Supplementary Material

Constructing the Sulfur-Doped CdO@In_2O_3 Nanofibers Ternary Heterojunction for Efficient Photocatalytic Hydrogen Production

Haiyan Zhang [1,†], Zi Zhu [1,†], Min Yang [1], Youji Li [1,*], Xiao Lin [1], Ming Li [1], Senpei Tang [1], Yuan Teng [1,*] and Dai-Bin Kuang [2,*]

[1] National Experimental Teaching Demonstration Center for Chemistry, College of Chemistry and Chemical Engineering, Jishou University, Jishou 416000, China; haiyanzhang2023@163.com (H.Z.); zhuzi7615@gmail.com (Z.Z.); yangmin18874346198@163.com (M.Y.); linxiao2017@whu.edu.cn (X.L.); liming25@foxmail.com (M.L.); chemtangjsu@163.com (S.T.)
[2] MOE Key Laboratory of Bioinorganic and Synthetic Chemistry, Lehn Institute of Functional Materials, School of Chemistry, Sun Yat-sen University, Guangzhou 510006, China
* Correspondence: jdyoujili@vip.163.com (Y.L.); tengy6@mail.sysu.edu.cn (Y.T.); kuangdb@mail.sysu.edu.cn (D.-B.K.)
† These authors contributed equally to this work.

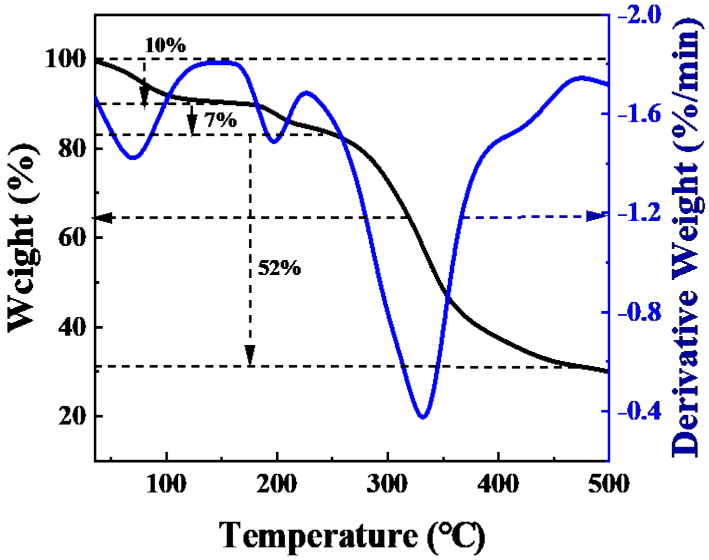

Figure S1. TG–DTA curves of S/CdO@In_2O_3–25 precursor fibers.

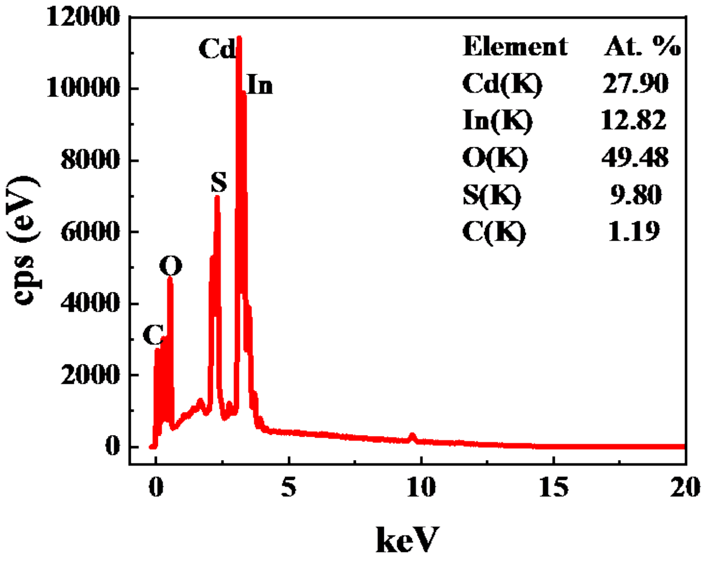

Figure S2. EDS spectrum of S/CdO@In2O3–25 nanofibers.

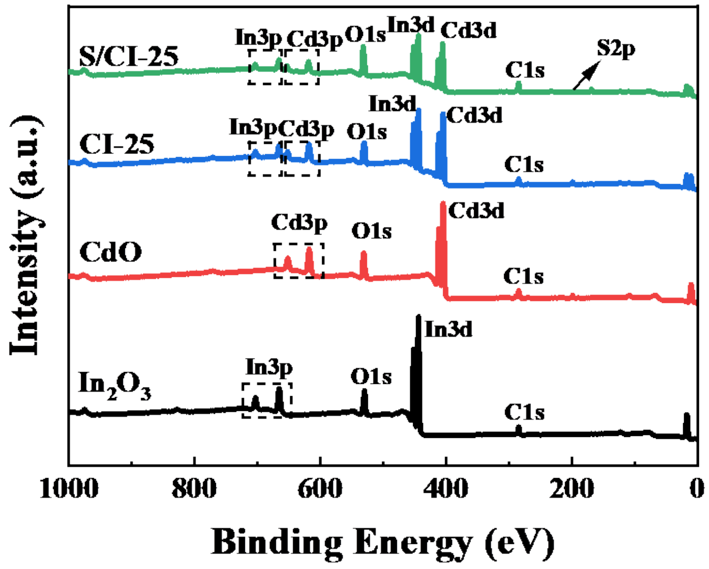

Figure S3. XPS survey spectra of the In2O3, CdO, CdO@In2O3–25 and S/CdO@In2O3.

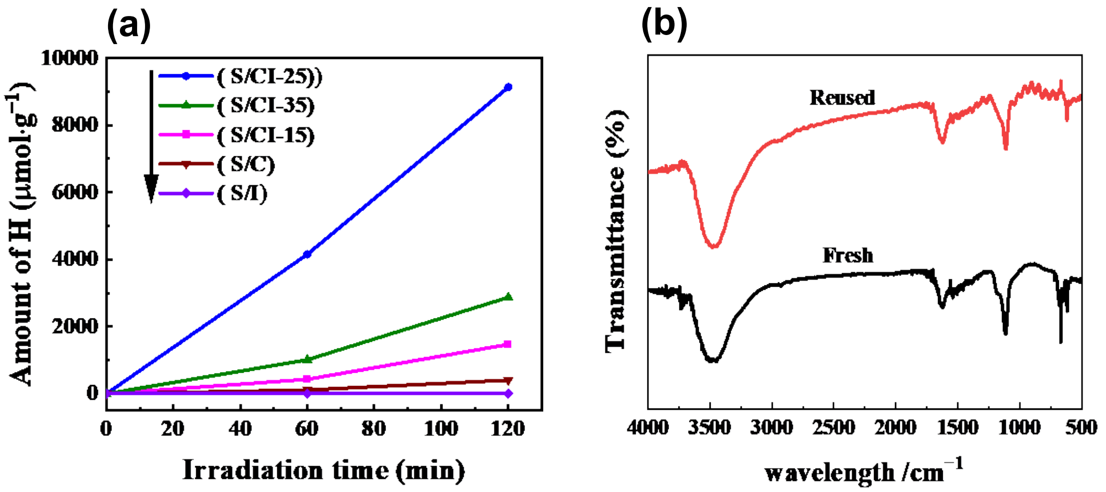

Figure S4. (**a**) The relationship between time and H_2 production amount over the synthesized photocatalysts. (**b**) FTIR spectra of S/CdO @ In_2O_3−25 catalyst before and after irradiation.

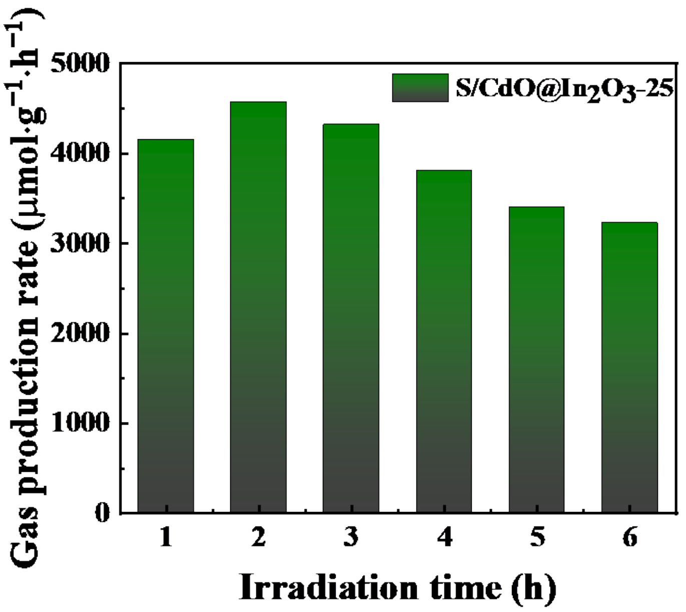

Figure S5. The relationship between reaction time and H_2 production amount over the S/CdO@In_2O_3−25.

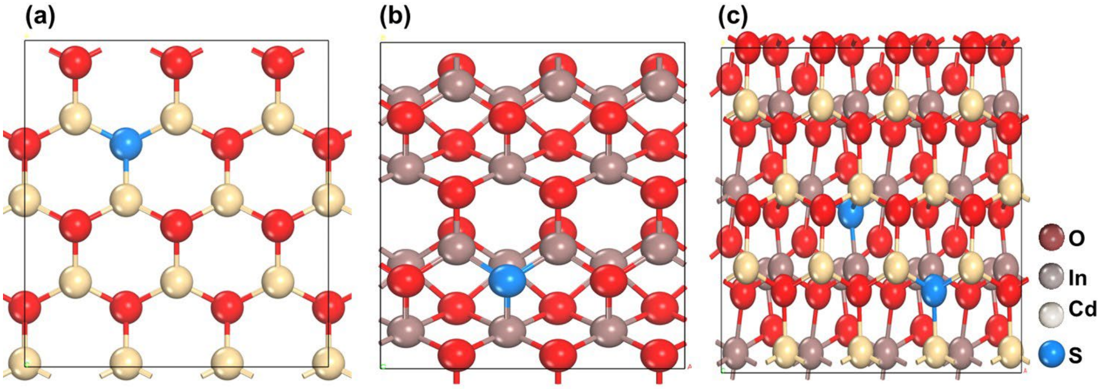

Figure S5. The geometrical structure of (**a**) S/CdO (**b**) S/In_2O_3 and (**c**) S/CdO@In_2O_3−25.

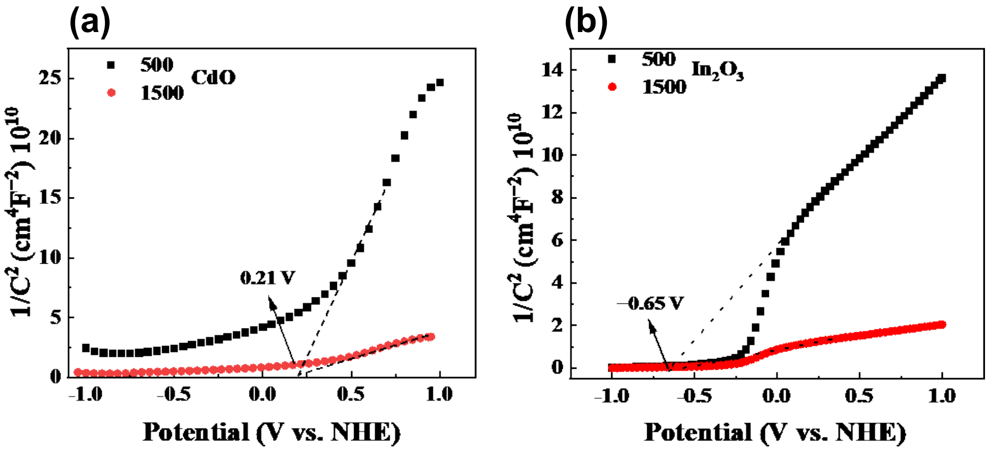

Figure S6. Mott−Schottky plots of (**a**) CdO and (**b**) In_2O_3.

Table S1. Comparison of HER performance for the partly reported CdO-based or In_2O_3-based photocatalysts in the literatures and this work.

Catalysts	Reaction medium	loading mass	Light source	H_2 yield rate (μmol·g^{-1}·h^{-1})	Ref.
CdS/In_2O_3	TEOA	-	300W Xe (λ>400nm)	235.05	[1]
$In_2S_3/CdIn_2S_4/In_2O_3$	Na_2SO_3 &Na_2S	1mg/ml	225 W Xe	2004	[2]
CdS-ZnO-CdO	Na_2SO_3 &Na_2S	-	150 W Xe	11.6	[3]
$In_2O_3/Au_4/CdS$-12	Na_2SO_3 &Na_2S	1wt%	225W Xe	830	[4]
CdO/g-C_3N_4	formaldehyde	-	100 W Xe	18.125	[5]
MOF/In_2O_3	TEOA	-	300 W Xe (λ≥400nm)	18.125	[6]
In_2O_3/C_3N_4	L-ascorbic acid	0.5wt%	300 W Xe (λ ⩾ 420nm)	197.5	[7]
TiO_2/g-C_3N_4	TEOA	1wt%	LED (λ≥400nm)	3211	[8]
TiO_2/g-C_3N_4	TEOA	1wt%	300 W Xe (λ≥420nm)	189	[9]
CTF-C_3N_4	TEOA +CH_3OH	5wt%	300 W Xe (λ≥420nm)	487.6	[10]
C/C_3N_4	TEOA	1wt%	300 W Xe (λ≥420nm)	560.8	[11]
S/CdO@In_2O_3-25	Na_2SO_3 &Na_2S	3wt%	300 W Xe (λ≥400nm)	4564.577	This work

Table S2. The energy band structure parameters of the samples.

Sample	E_g	E_f	E_{CB}	E_{VB}
CdO	1.94	0.21	0.11	2.05
S/CdO	2.39	-0.51	-0.61	1.78
In_2O_3	2.78	-0.65	-0.75	2.03
S/In_2O_3	2.54	-0.70	-0.8	1.74

References

1. Ren, J.-T.; Yuan, K.; Wu, K.; Zhou, L.; Zhang, Y.-W. A robust CdS/In_2O_3 hierarchical heterostructure derived from a metal-organic framework for efficient visible-light photocatalytic hydrogen production. *Inorg. Chem. Front.* **2019**, *6*, 366–375.
2. Ma, D.; Shi, J.W.; Zou, Y.; Fan, Z.; Shi, J.; Cheng, L.; Sun, D.; Wang, Z.; Niu, C. Multiple carrier-transfer pathways in a flower-like In_2S_3/$CdIn_2S_4$/In_2O_3 ternary heterostructure for enhanced photocatalytic hydrogen production. *Nanoscale* **2018**, *10*, 7860–7870.
3. Navarro, R.; Delvalle, F.; Fierro, J. Photocatalytic hydrogen evolution from CdS-ZnO-CdO systems under visible light irradiation: Effect of thermal treatment and presence of Pt and Ru cocatalysts. *Int .J. Hydrogen Energ*. **2008**, *33*, 4265–4273.
4. Ma, D.; Shi, J.-W.; Sun, D.; Zou, Y.; Cheng, L.; He, C.; Wang, Z.; Niu, C. Au Nanoparticle and CdS Quantum Dot Codecoration of In_2O_3 Nanosheets for Improved H_2 Evolution Resulting from Efficient Light Harvesting and Charge Transfer. *ACS Sustain.Chem. Eng.* **2018**, *7*, 547–557.
5. Munusamy, T.D.; Chin, S.Y.; Tarek, M.; Khan, M.M.R. Sustainable hydrogen production by CdO/exfoliated g-C_3N_4 via photoreforming of formaldehyde containing wastewater. *Int. J. Hydrogen Energ.* **2021**, *46*, 30988–30999.
6. Han, L.; Jing, F.; Zhang, J.; Luo, X.-Z.; Zhong, Y.-L.; Wang, K.; Zang, S.-H.; Teng, D.-H.; Liu, Y.; Chen, J.; et al. Environment friendly and remarkably efficient photocatalytic hydrogen evolution based on metal organic framework derived hexagonal/cubic In_2O_3 phase-junction. *Appl. Catal. B-Environ.* **2021**, *282*, 119602.
7. Cao, S.-W.; Liu, X.-F.; Yuan, Y.-P.; Zhang, Z.-Y.; Liao, Y.-S.; Fang, J.; Loo, S.C.J.; Sum, T.C.; Xue, C. Solar-to-fuels conversion over In_2O_3/g-C_3N_4 hybrid photocatalysts. *Appl. Catal. B-Environ.* **2014**, *147*, 940–946.
8. Yang, J.; Wu, X.; Mei, Z.; Zhou, S.; Su, Y.; Wang, G. CVD Assisted Synthesis of Macro/Mesoporous TiO_2/g-C_3N_4 S-Scheme Heterojunction for Enhanced Photocatalytic Hydrogen. *Evol. Adv. Sustain. Syst*. **2022**, *6*, 220005.
9. Zhu, H.; Yang, X.; Zhang, M.; Li, Q.; Yang, J. Construction of 2D/2D TiO_2/g-C_3N_4 nanosheet heterostructures with improved photocatalytic activity. *Mater. Res. Bull.* **2020**, *125*, 110765.
10. Gong, R.; Yang, L.; Qiu, S.; Chen, W.-T.; Wang, Q.; Xie, J.; Waterhouse, G.I.N.; Xu, J. A Nitrogen-Rich Covalent Triazine Framework as a Photocatalyst for Hydrogen Production. *Adv. Polym. Tech.* **2020**, *2020*, 7819049.
11. Xiao, M.; Jiao, Y.; Luo, B.; Wang, S.; Chen, P.; Lyu, M.; Du, A.; Wang, L. Understanding the roles of carbon in carbon/g-C_3N_4 based photocatalysts for H_2 evolution. *Nano Res.* **2021**, https://doi.org/10.1007/s12274-021-3897-7.
